# The socio-cultural importance of *Mauritia flexuosa* palm swamps (aguajales) and implications for multi-use management in two Maijuna communities of the Peruvian Amazon

**DOI:** 10.1186/1746-4269-9-29

**Published:** 2013-04-22

**Authors:** Michael P Gilmore, Bryan A Endress, Christa M Horn

**Affiliations:** 1 New Century College, George Mason University, 4400 University Drive, MS 5D3, Fairfax, VA 22030, USA; 2 Division of Applied Plant Ecology, Institute for Conservation Research, San Diego Zoo Global, 15600 San Pasqual Valley Road, Escondido, CA, 92027, USA; 3 Division of Applied Plant Ecology, Institute for Conservation Research, San Diego Zoo Global, 15600 San Pasqual Valley Road, Escondido, CA, 92027, USA

**Keywords:** Ethnoecology, Multi-use management, Forest resources, Maijuna, Peruvian Amazon, *Mauritia flexuosa*

## Abstract

**Background:**

Fruit from the palm *Mauritia flexuosa* (aguaje) is harvested throughout the Peruvian Amazon for subsistence and commercial purposes. Recent estimates suggest that residents of Iquitos, the largest city in the region, consume approximately 148.8 metric tons of aguaje fruit per month, the vast majority of which is harvested by felling and killing adult female trees. In this study, we sought to better understand and document the importance of *M. flexuosa* palm swamps (aguajales) in two Maijuna indigenous communities to inform the sustainable management of this habitat and species.

**Methods:**

Semi-structured interviews, focus groups, and household surveys were carried out to assess the significance of aguajales and their associated plant and animal resources as well as to determine how the relationship that the Maijuna have with aguajales has changed over time.

**Results:**

Aguajales and their associated resources are culturally significant and useful to the Maijuna in a wide variety of ways. In addition to *M. flexuosa*, the Maijuna use over 60 different species of plants from aguajales. When *M. flexuosa* is in fruit, aguajales are important hunting areas with a total of 20 different animal species hunted. The Maijuna also have traditional beliefs about aguajales, believing that malevolent supernatural beings reside in them. Notably, the relationship that the Maijuna have with aguajales has changed considerably over the years as aguaje fruit went from a subsistence item collected opportunistically from the ground to a market good destructively harvested beginning in the early 1990s. The Maijuna are concerned not only about how this has affected the future commercial harvest of aguaje but also about its effects on game animals given the importance of hunting to Maijuna cultural identity, subsistence, and income generation.

**Conclusions:**

In order to meet the multiple socio-cultural and economic needs of the Maijuna, sustainable management efforts must be expanded to not only focus on the commercial harvest of aguaje but also other facets of their relationship with this habitat. Our study suggests that the research and development of multi-use forest management plans must not be restricted to commercial forest products and ecosystem services given that many communities rely on tropical forests for a wide range of non-market cultural, economic, and subsistence goods and services.

## Abstract (es)

**Resumen:**

**Antecedentes**

La fruta de la palma *Mauritia flexuosa* (aguaje) se cosecha en toda la Amazonía peruana con fines de subsistencia y comerciales. Recientes estimados sugieren que los habitantes de Iquitos, la ciudad más grande en la región, consumen aproximadamente 148.8 toneladas métricas de fruta mensualmente, la gran mayoría de las cuales se cosecha mediante la tala de palmas adultas de genero femenino. En este estudio, hemos tratado de comprender y documentar la importancia de los pantanos de las palmeras *M. flexuosa* (aguajales) en dos de las comunidades indígenas Maijuna y de esta manera informar el manejo sostenible de este hábitat y esta especie.

**Métodos**

Entrevistas semiestructuradas, grupos focales y encuestas a hogares se llevaron a cabo para evaluar la importancia de aguajales y los recursos de plantas y animales asociados a estos. Así como también para determinar cómo la relación que tienen los Maijuna con los aguajales ha cambiado a través del tiempo.

**Resultados**

Los aguajales y los recursos asociados con estos son culturalmente significativos y útiles para los Maijuna en una amplia variedad de formas. Adicionalmente a *M. flexuosa*, los Maijuna utilizan más de 60 diferentes especies de plantas en los aguajales. Cuando la cosecha de fruta de *M. flexuosa* ocurre, los aguajales son importantes áreas de caza ya que un total de 20 especies diferentes de animales pueden ser casados. Los Maijuna también tienen creencias tradicionales relacionadas a los aguajales, existe una creencia que seres sobrenaturales malévolos residen en allí. Es de destacar que la relación que tienen los Maijuna con los aguajales ha cambiado considerablemente a través del tiempo, ya que el fruto de aguaje pasó de ser un elemento de subsistencia recolectado de manera oportunista de la tierra, a un bien de mercado cosechado de manera destructiva desde principios de los 1990. Los Maijuna están preocupados no sólo sobre cómo esta practica ha afectado el futuro de la cosecha comercial del aguaje, sino también sobre sus efectos en los animales de caza, dada la importancia que tienen la caza en su identidad cultural y su subsistencia y generación de ingresos.

**Conclusiones**

Con el fin de satisfacer las múltiples necesidades socio-culturales y económicas de los Maijuna, los esfuerzos de manejo sostenible deben expandirse a enfocar no sólo a la cosecha comercial de aguaje, sino también tener en cuenta otras facetas de su relación con este hábitat. Dado que muchas comunidades dependen de los bosques tropicales en una amplia gama de aspectos culturales, económicos y de subsistencia que no están relacionados al mercado, nuestro estudio sugiere que la investigación y el desarrollo de los planes de usos múltiples para el manejo forestal no deben limitarse únicamente a los productos forestales comerciales y a los servicios de los ecosistemas.

## Background

Throughout many parts of the Amazon basin, fruit from the palm *Mauritia flexuosa* L.f. is harvested for subsistence and commercial purposes. Known as *aguaje* in the Peruvian Amazon, the commercial extraction of fruit from this dioecious palm provides an important source of income for rural communities [1,2] as well as urban families living in and near the city of Iquitos [3]. The fruit is consumed raw or processed into a variety of products (e.g. beverages, ice cream, ice pops, etc.) and recent estimates suggest that residents of Iquitos consume approximately 148.8 metric tons of aguaje fruit per month, the vast majority of which is harvested by the felling and killing of adult female trees in the forest [4].

The consistent demand for *M. flexuosa* and the destructive nature of the harvest has resulted in serious over-exploitation and degradation of naturally occurring *M. flexuosa*-dominated palm swamps (known as *aguajales*; [2,5]). Destructive harvesting results in skewed sex ratios, with over-harvested stands dominated by male individuals [2,6]. Not only does destructive harvest undermine the palm’s economic potential for rural communities, but it also likely disrupts a number of ecological patterns and processes. Fruit from *M. flexuosa* is an important food source for a wide range of wildlife [7-9], while a number of bird species (e.g. macaws) nest in cavities of standing dead individuals [10,11].

In response to over-exploitation of *M. flexuosa* and the degradation of aguajales, recent resource management efforts have focused on sustainable harvesting techniques (via non-destructive climbing), cultivation, and agroforestry systems [1,2,12]. Numerous factors have been shown to influence adoption rates of these approaches and the implementation of sustainable harvest plans, including access to climbing devices and training, organizational experience, low female abundance (due to previous felling), and market barriers [1,2]. Many of these factors synergistically interact to hinder the widespread incorporation of sustainable harvest techniques in the Peruvian Amazon, and the vast majority of aguaje fruit continues to be destructively harvested for the commercial market.

Like many communities, the Maijuna, an indigenous group inhabiting several river basins in Loreto, Peru, are interested in halting destructive harvest of *M. flexuosa* and developing aguaje management plans. While the development of a sustainable aguaje harvest and management plan for fruit extraction appears relatively straightforward in concept (i.e. eliminate destructive harvest, provide training and capacity building to harvest non-destructively, etc.), management plans focused solely on the production of aguaje fruit for the commercial market may be inadequate as the Maijuna rely on their ancestral forests, including aguajales, for a wide range of goods and services for multiple economic, subsistence, and cultural purposes [13]. In order to effectively develop management plans for aguaje and aguajales in Maijuna lands it is critical to properly understand their significance to the Maijuna and how they holistically interact with this species and habitat.

Multiple-use forest management has received considerable attention in recent years, though integrated approaches to forest management, particularly when involving non-timber forest products in the tropics, remain poorly evaluated [14,15]. Of the studies that have been conducted the vast majority have largely focused on managing for the extraction of multiple forest products for commercial purposes (e.g. timber and market non-timber forest product extraction). Yet, many communities rely on tropical forests for a much wider range of cultural, economic, and subsistence goods and services; therefore the development of culturally relevant natural resource management plans must not be restricted to simply commercial forest products, but also incorporate non-market goods and services. The objective of this study was to document and understand the importance of aguaje and aguajales to Maijuna culture and livelihoods in order to inform the sustainable harvest and management of this species and habitat and ensure plans incorporate and account for the multiple cultural and economic needs of the Maijuna people. Specifically, we: (1) assessed the significance of aguajales and their associated plant and animal resources to the Maijuna and (2) documented changing uses and relationships between the Maijuna and aguajales over the past century.

### Aguaje and aguajales

*Mauritia flexuosa* is a long-lived, arborescent, and dioecious palm found throughout wetland and swamp habitats of the lowland tropics of South America. It is common in the Amazon Basin where it is often dominant or co-dominant in naturally occurring swamp forests (aguajales) located in floodplains of rivers and streams or in poorly drained shallow depressions in upland forest that are flooded only by rains [16]. Heights of *M. flexuosa* can exceed 30 m and leaves can measure up to 2.5 m long and 4.5 m wide. Inflorescences up to 2 m in length emerge from between petioles and support 25-40 branches of flowers. In females, the inflorescences become pendulous with fruit. The fruits are up to 7 cm long and 5 cm in diameter [17]. In the Peruvian Amazon near Iquitos, fruiting peaks between July and September [5], though the fruiting season varies throughout its range. Aguajales play several important ecological roles by providing habitat and food resources to wildlife [7-11,17]. Additionally, they store a significant amount of carbon within their waterlogged soils and thick layers of organic matter underscoring the importance of these areas for carbon storage [18].

### The Maijuna

**Figure 1:**
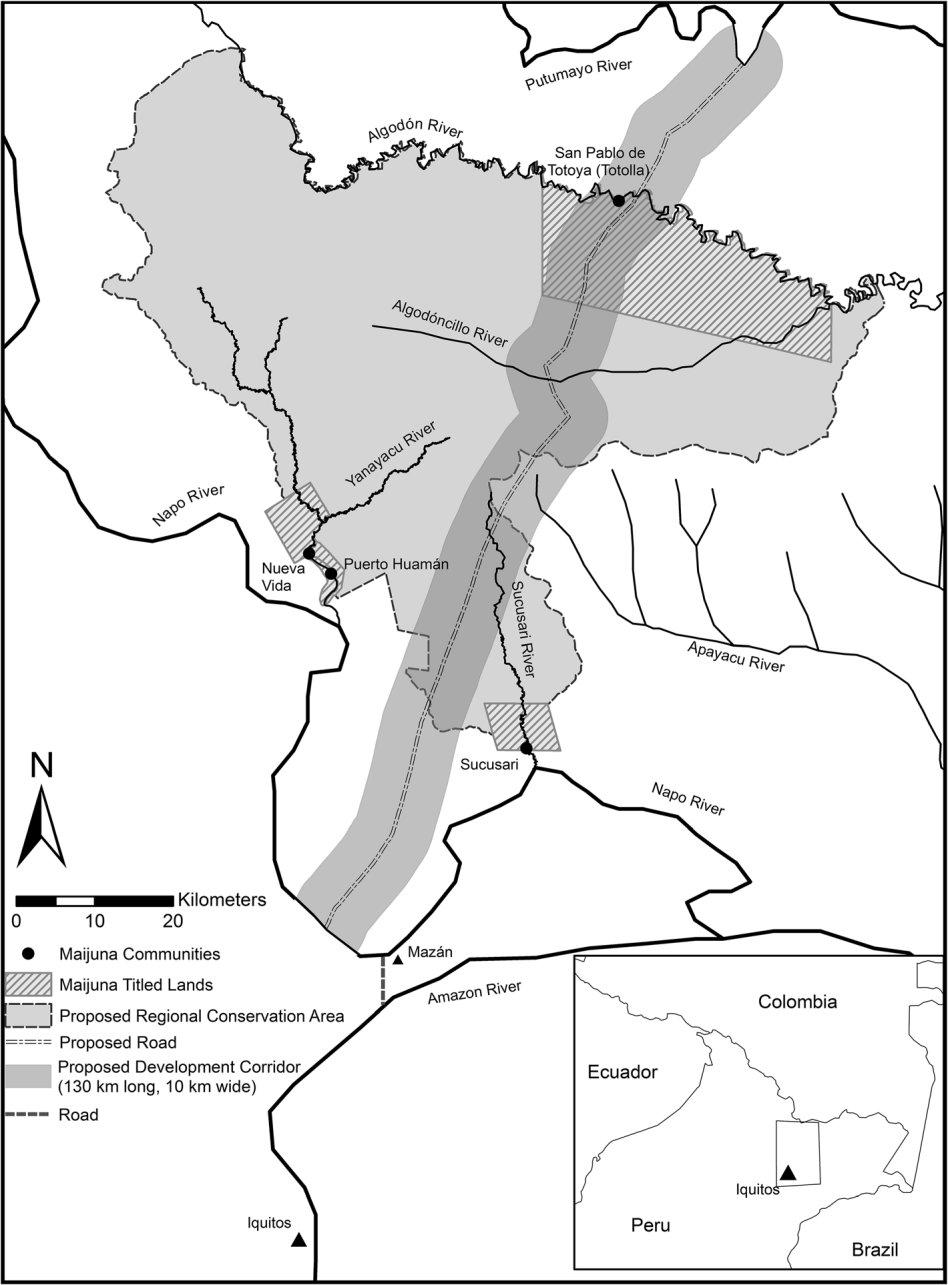
**Map of the study area, including the four Maijuna communities, the proposed road, proposed development corridor, and proposed regional conservation area.** All field research was conducted in the Maijuna communities of Nueva Vida and Puerto Huamán.

The Maijuna, also known as the Orejón, are a western Tucanoan people of the northeastern Peruvian Amazon [19-21]. Approximately 400 Maijuna individuals live in four communities along the Yanayacu, Sucusari, and Algodón rivers (Figure 1). These three river basins are part of the ancestral territory of the Maijuna [13]. The Maijuna traditionally lived in the interfluvial area between these three rivers, a practice lasting until the early 1900s when the influence of missionaries and *patrones*^a^ prompted the Maijuna to slowly migrate downriver to where they eventually formed their current communities [20,21]. The building of schools and the Maijuna desire to be in better contact with outside communities and services have served to maintain current settlement patterns.

Maijuna communities are composed of mono- and pluri-familial houses that are arranged according to kin ties, which exchange products and services. Families employ a variety of subsistence and income generating strategies, including swidden-fallow agriculture, hunting, fishing, and the gathering of various forest products. Each of the four Maijuna communities is recognized as a *Comunidad Nativa* (Native Community) by the Peruvian Government and has been granted title to land surrounding their community [22]. However, the titled land that the Maijuna communities have received is a small portion of their ancestral territory in the Yanayacu, Sucusari, and Algodón river basins and therefore hundreds of thousands of hectares of land remain outside of direct Maijuna control [13]. Additionally, Maijuna ancestral lands are under threat from poaching and logging from outsiders. Even more serious, the Peruvian government is pushing to build a 130 km long road, with a 5 km development corridor on either side of it, directly through the heart of Maijuna ancestral lands [23]. This proposed road and development corridor, along with a subsequent influx of colonists into the area, would irreversibly alter the ecological fabric of this currently roadless area and negatively impact Maijuna livelihood strategies and their current way of life.

In 2004, in response to these threats and challenges, Maijuna elders and leaders established the *Federación de Comunidades Nativas Maijuna* (FECONAMAI), an indigenous federation representing all four Maijuna communities [13]. Since its inception, the principle goals of FECONAMAI are to: (1) conserve the environment, (2) conserve the Maijuna culture, and (3) improve Maijuna community organization. Since 2008, FECONAMAI has been calling on the *Gobierno Regional de Loreto* (GOREL; the regional government of the Peruvian Amazon) to create an *Área de Conservación Regional* (ACR; regional conservation area) that would formally and legally protect over 336,000 hectares of the ancestral land to which they lack title (Figure 1) [23]. In 2012, GOREL approved the creation of the Maijuna ACR, and the proposal is now being reviewed by the national government.

## Methods

### Study site

Fieldwork was conducted over two field seasons during August 2010 and May 2011 in the Maijuna communities of Puerto Huamán and Nueva Vida, located along the Yanayacu River in the northeastern Peruvian Amazon (Figure 1). The communities of Puerto Huamán and Nueva Vida have populations of 95 and 109 people, respectively. The two communities are 4 km apart along the Yanayacu River, which is 220 km by river from Iquitos, the commercial center and largest city in the region. This trip is shortened to 95 km by crossing the thin isthmus between the Napo and Amazon Rivers by road at the small port town of Mazán.

The Yanayacu River basin is a relatively flat area, similar to the rest of the Peruvian Amazon, with an elevation varying from 80-200 m above sea level [24]. The area includes both upland and floodplain forests. Aguajales within the Yanayacu River basin are found both in upland forest depressions flooded by rains and in the floodplains of rivers and streams. This region of the Peruvian Amazon is tropical, humid, and warm, having a mean annual precipitation of approximately 3100 mm per year and a mean annual temperature of 26°C [25].

### Data collection and analysis

Before beginning research, prior informed consent (PIC) was obtained from each of the communities as well as from research participants [26]. During the course of this study, we conducted focus groups and semi-structured interviews with community leaders and cultural specialists regarding the use, classification, and significance of aguajales and their associated plant and animal resources as well as the changing relationship between the Maijuna and aguajales over the past century. Approximately one dozen male and female Maijuna individuals, 35 years in age and older, participated in this portion of the research. To further understand these topics and delve deeper into current patterns of aguaje harvest, we also conducted structured surveys with the heads of households, or their spouses, in both communities. All 20 households in Nueva Vida and 16 out of 17 households in Puerto Huamán were surveyed. All of this information was supplemented by data that one of the authors (Gilmore) has collected regarding the use and significance of aguaje and aguajales by the Maijuna since 1999 (e.g. see [27]). To identify key themes, perceptions, and issues qualitative data were coded, organized, and analyzed based on the methods described by Strauss and Corbin [28].

Additionally, to identify culturally significant and useful plant species found in aguajales, we established forest survey plots in 12 aguaje stands near the two communities. Stands of varying accessibility to the communities were selected using Maijuna guides and previously completed participatory maps of the area (see [29,30]). All stands were located in poorly drained depressions in upland forest that are inundated only by rainfall and all were within one half-day of travel (by foot and/or boat) from the communities. We focused on aguajales in poorly drained depressions in upland forest because they are more widely distributed throughout the Yanayacu River basin and more heavily used by the Maijuna of both communities as compared to aguajales in floodplains of rivers and streams. Plots were established in each stand and consisted of three circular subplots, each with 10.3 m radii, which resulted in an area sampled of 0.1 ha per stand. Subplots were spaced a minimum of five meters apart and no plot was within 10 m of the edge of an aguajal. Maijuna individuals (six men ages 30-57) well known for their knowledge of plants then identified all useful plant species found in each plot. Ethnobotanical data regarding the Maijuna name, local name, use, harvesting method, and time of harvest was collected for each useful species. All of this information was supplemented by ethnobotanical data that one of the authors (Gilmore) has collected since 1999 from interviewing dozens of adult Maijuna male and female community members and cultural specialists (e.g. see [27]). It is important to note that voucher specimens were collected and deposited in the Herbarium Amazonense (AMAZ), Universidad Nacional de la Amazonia Peruana, Iquitos, Peru.

## Results and discussion

### Classification of aguajales and aguaje

The Maijuna have an extensive and complex habitat classification system for both forest and non-forest habitats found within their ancestral lands [27]. Their habitat classification system is not a perfectly hierarchical system. Instead, it is composed of multiple, separate overlapping sub-systems that they use to classify habitats. They classify habitats based on geomorphology, physiognomy, disturbance, indicator plant species, and indicator animal species. Of particular interest to this study is the fact that the Maijuna recognize and name habitats defined by indicator plant species that are located in swampy areas [27]. All of the Maijuna names for these habitats are formed by joining the name of the indicator plant species with the Maijuna word ***cuadu***^b^ which literally means ‘soft earth’. The Maijuna name for a *M. flexuosa* palm swamp or aguajal is ***ne cuadu*** which can be literally translated as ‘*M. flexuosa* in soft earth’. The Maijuna recognize ***ne cuadu*** that are located both in floodplains and poorly drained upland forests which ultimately corresponds to the Western ecological description of this habitat. In short, *M. flexuosa* palm swamps are habitats that are both culturally defined and recognized by the Maijuna. Notably, this is also the case as well in other indigenous (e.g. see [31,32]) and non-indigenous communities throughout the Peruvian Amazon.

According to the Maijuna, three different varieties of *M. flexuosa* (***ne ñi***) are found growing in ***ne cuadu*** within Maijuna lands and they are classified based on the color of their fruit pulp: ***ma ne*** (‘red aguaje’), ***sɨño ne*** (‘yellow aguaje’), and ***bo ne*** (‘white aguaje’). These three Maijuna recognized varieties of aguaje are also distinguished locally and regionally within the Spanish vernacular, with ***ma ne*** being *aguaje shambo*, ***sɨño ne*** being *aguaje amarillo* and ***bo ne*** being *aguaje posheco*. ***Ma ne*** or *aguaje shambo* has the most red and oily pulp of the three varieties. ***Sɨño ne*** and ***bo ne*** are different shades of yellow, with ***sɨño ne*** being a stronger yellow color and ***bo ne*** being pale yellow in color instead of pure white as the Maijuna name may suggest.

### Use and significance of aguajales and aguaje

*Mauritia flexuosa* palm swamps (***ne cuadu***) are the largest and most culturally important habitat defined by indicator plant species located in areas with ‘soft earth’. Its namesake species, *M. flexuosa* (***ne ñi***), is used by the Maijuna for a wide variety of major and minor ethnobotanical uses (Table 1). However, the most important plant product obtained from this tree is its fruit. The fruit are eaten, made into a beverage, processed into an oil, and used as fishing bait. The fruits are also economically important, due to their value in the regional economy [2,3,5,33]. Within the Yanayacu River basin, *M. flexuosa* fruits from approximately May to August and during this time ***ne cuadu*** become important fruit collecting areas. ***Ma ne*** (*aguaje shambo*) is the most prized of the three Maijuna recognized aguaje varieties and garners the highest price on the regional market, though fruit from both ***sɨño ne*** and ***bo ne*** are also harvested, consumed, and sold.

**Table 1:** **Ethnobotanical information corresponding to useful plant species found in *M. flexuosa *** **palm swamps ( *ne cuadu *) within the Yanayacu River basin**

**Taxon [voucher]**^**a**^		**Maijuna name**	**Spanish name**	**Use**	**Harvesting method**	**Time of harvest**^**b**^
Annonaceae						
	*Annona *sp. 1^c ^[668]	***aña mica ñi***	anonilla	fruits: edible	picked	unknown
	*Duguetia *sp. 1^c ^[715]	***yai j *** ***ɨ*** ***tɨ ada ñi****,* ***ɨ*** ***sɨbo ñi***	tortuga caspi	trunk: house construction material	felled	year round
				trunk: firewood	felled	year round
	*Guatteria decurrens *R.E. Fr. [648]	***nea cañi ***(‘black strap tree’)	carahuasca negra	trunk: house construction material	felled	year round
				bark: strips used as a strapping to carry things	stripped from felled tree	year round
	*Oxandra euneura *Diels [685]	***ai codiyo ñi ***(‘old rib tree’), ***bitoyo ñi ***(‘fishing pole tree’)	tortuga caspi	trunk: house construction material	felled	year round
				trunk: treelets used as fishing poles	felled	year round
	*Unonopsis guatterioides* R. E. Fr. [664]	***nea cañi ***(‘black strap tree’)	carahuasca negra	trunk: house construction material	felled	year round
				bark: strips used as a strapping to carry things	stripped from felled tree	year round
	*Unonopsis peruviana* R. E. Fr. [704]	***nea cañi ***(‘black strap tree’)	carahuasca negra	trunk: house construction material	felled	year round
				bark: strips used as a strapping to carry things	stripped from felled tree	year round
Apocynaceae						
	*Aspidosperma *sp. 1^c ^[666]	***yototo ñi ***(‘canoe buttress tree’)	remo caspi	buttress roots: used to make canoe paddles and ax handles	cut from buttress root (not felled)	year round
				trunk: firewood	felled	year round
	*Aspidosperma *sp. 3^c ^[673]	***sɨño come toto ***(‘yellow paddle buttress tree’)	remo caspi amarillo	buttress roots: used to make canoe paddles and ax handles	cut from buttress root (not felled)	year round
				trunk: firewood	felled	year round
	*Himatanthus sucuuba *(Spruce ex Müll. Arg.) Woodson [776]	***dodo ñi***	bellaco caspi	latex: medicinal (abscesses/boils)	tap trunk	year round
	*Parahancornia peruviana *Monach. [692]	***s *** ***ɨ*** ***e*** ***ca ñi***	naranjo podrido	fruits: edible	collected from felled tree	~April-July
Araceae						
	*Dracontium *sp. 1^c ^[724]	***aña cajo*** (‘snake’s tuber’)	jergón sacha	tuber: medicinal (used to treat snake bites)	extracted from ground	year round
				leaf/petiole: medicinal/traditional beliefs (used to prevent snake bites)	cut from plant	year round
Arecaceae						
	*Astrocaryum chambira *Burret [767]	***beto ñi****,* ***ñuca ñi***	chambira	fruits: edible (toast and eat mature fruits and eat liquid/spongy endosperm of immature fruits)	collected from ground, by using pole, or felling tree	~January-March
				spear leaf: fiber extracted from immature pinnae used to make handicrafts (hammocks, bags, baskets, etc.); handicrafts sold	cut from plant (plant not felled except when tall)	year round
				spear leaf: midrib of immature pinnae used to make brooms	same as above	same as above
				spear leaf: remaining portions of immature pinnae used in basket making after removal of fibers and midribs; baskets sold	same as above	same as above
				spear leaf: small immature pinnae toward top of spear leaf used to make fans for tourist trade and fanning fires; fans sold	same as above	same as above
	*Astrocaryum macrocalyx* Burret [782]	***chida ñi***	huicungo	fruits: edible (liquid/spongy endosperm)	collected from felled tree	unknown
				sprouting seeds: medicinal oil (pimples)	collected from ground	year round
				trunk: construction material (house and floor support posts)	felled	year round
				trunk: pry bars for canoe construction	felled	year round
				seeds: seed coat used to adorn ear disks^d^	collected from ground	year round
				spear leaf: immature leaflets used to make “crowns” and “flags” for special occasions and traditional ceremonies	cut from plant (harvested from small plants)	year round
	*Attalea insignis* (Mart. ex H. Wendl.) Drude [763]	***edi ñi***	inayuga, shapaja	fruits: edible	picked	year round
				petioles: used to stretch animal hides during the drying process	cut from plant	year round
				petioles: used to make blowgun darts^d^	portion cut from petiole (leaves not removed)	year round
	*Attalea maripa* (Aubl.) Mart. [778]	***edi ñi***	shapaja	fruits: older fruits host beetle larvae that are eaten and used as fishing bait	collected from ground	year round
				fruits: edible	collected from ground, by climbing leaning pole, or felling tree	year round
				leaves: thatch for houses	collected from felled tree	year round
				leaves: thatch for the ridges of roofs	cut from plant (harvested from small plants)	year round
				spathe: used as a dish to store things and as a child’s toy canoe	collected from ground	year round
				seeds: used to smooth and/or polish clay during the production of ceramics	collected from ground	year round
	*Bactris maraja *Mart.	***bi ñi***	chontilla	fruits: edible	picked	~March-April
	*Desmoncus mitis* Mart. [781]	***jijebɨ meme ***(‘sieve vine’)		stem: used to lash together the frames of sieves	stems cut from plant	year round
	*Desmoncus polyacanthos *Mart.	***jijebɨ meme ***(‘sieve vine’)		stem: used to lash together the frames of sieves	stems cut from plant	year round
	*Euterpe precatoria *Mart. [313^e^, 531^e^]	***ɨ*** ***mɨbi ñi, ɨ mɨ bɨe ñi***	huasai, chonta	fruits: edible (used to make a beverage); rarely sold	collected from felled tree	year round
				leaves: thatch for temporary shelters	cut from plant (plant not felled except when tall)	year round
				palm heart: edible; sold	extracted from felled tree	year round
				roots: processed into a medicine (malaria)	extracted from ground (not felled)	year round
				trunk: construction material (house railings and walls)	felled	year round
				crown shaft: used to package processed blocks of *Couma macrocarpa* latex^d^	extracted from felled tree	year round
	*Geonoma deversa* (Poit.) Kunth	***n *** ***i*** ***n *** ***i*** ***ñi***	palmicha	leaves: occasionally (when abundant) placed on the ground to quarter animals while hunting	cut from plant	year round
				leaves: thatch for temporary shelters	cut from plant	year round
	*Geonoma macrostachys *Mart. var. *acaulis *(Mart.) Skov [762]	***n *** ***i*** ***n *** ***i*** ***ñi***		leaves: occasionally (when abundant) placed on the ground to quarter animals while hunting	cut from plant	year round
	*Mauritia flexuosa *L. f. [321^e^, 529^e^]	***ne ñi***	aguaje	fruits: edible (eaten, used to make a beverage, and processed into an oil); sold	collected from ground and by climbing or felling tree	~May-August
				fruits: pieces used as fishing bait	same as above	same as above
				leaves: use old, dry leaves as a fuel for drying canoes and starting fires in newly cleared and dried agricultural fields	old and hanging leaves cut off of tree	year round
				petioles: strips of fiber used to make mats and used as a form for weaving palm fiber bags	cut from plant (harvested from small plants)	year round
				trunk: hosts two species of beetle larvae that are eaten and used as fishing bait	from trees felled to promote larval growth and natural tree falls	year round
	*Mauritiella armata *(Mart.) Burret	***bɨe ne ñi***	aguajillo	fruits: edible	collected from ground or by felling tree	~May-August
	*Oenocarpus bataua *Mart. [324^e^, 555^e^]	***bosa ñi****, ****osa ñi****, ****g *** ***o*** ***sa ñi***	hunguraui, unguraui	fruits: edible (eaten, used to make a beverage, and processed into an oil); occasionally sold	collected from ground and by climbing or felling tree	~November-March and June-August
				fruits (unripe): processed into a medicine (tuberculosis)	collected by climbing or felling tree	~year round
				leaves: used to make temporary baskets	cut from plant (harvested from small plants)	year round
				leaves: thatch for temporary shelters	cut from plant (plant not felled except when tall)	year round
				trunk: hosts a beetle larva that is eaten and used as fishing bait	from trees felled to promote larval growth and natural tree falls	year round
				leaf base fibers: sharpened and used to pierce men’s ears for ear disks^d^	collected from plant (plant not felled)	year round
				leaf base fibers: used as kindling^d^	collected from felled tree	year round
	*Oenocarpus mapora *H. Karst. [780]	***bi bosa ñi, bi osa ñi, bi g osa ñi ***(‘small *Oenocarpus bataua *tree’)	cinamillo	fruits: edible (eaten and used to make a beverage)	collected by climbing or felling tree	~November-March and June-August
				leaves: used to make temporary baskets	cut from plant (harvested from small plants)	year round
				leaves: thatch for temporary shelters	cut from plant (plant not felled except when tall)	year round
				petioles: strips of fiber used to make sieves	cut from plant (harvested from small plants)	year round
				trunk: construction material (support posts for small structures)	felled	year round
	*Socratea exorrhiza *(Mart.) H. Wendl. [315^e^, 530^e^]	***j *** ***ɨ*** ***co ñi***	cashapona	stilt roots: spiny sections used as graters	cut from stilt root (not felled)	year round
				trunk: construction material (floors of houses and temporary shelters; walls of houses; slats also used to weave thatch around); occasionally sold	felled	year round
				trunk: used to make platforms above cooking fires to dry and smoke food	felled	year round
				trunk: used to make spears for hunting and warfare^d^	felled	year round
Burseraceae						
	*Protium *spp. [716]	***bayidi ñi***	copal	resin balls: used to seal/caulk canoes, etc.	picked from tree (not felled)	year round
				resin balls: used as a fuel to start fires	same as above	same as above
				resin balls: used as a fuel for a type of candle^d^	same as above	same as above
Chrysobalanaceae						
	*Licania heteromorpha *Benth. [718, 736]	***cobe aoñi ***(‘*Eira barbara*’s food tree’)		fruits: edible	collected from felled tree	unknown
	*Parinari parilis *aff. J.F. Macbr. [655]	***mateto ñi***	parinari	fruits: edible	collected from ground	~October-November and January-March
	*Parinari *sp. 1^c ^[706]	***mateto ñi***	parinari	fruits: edible	collected from ground	~October-November and January-March
Clusiaceae						
	*Chrysochlamys ulei *Engl. [757]	***ñase sada ñi***		trunk: firewood	felled	year round
	*Symphonia globulifera *L. f. [743]	***maja ñi ***(‘tar tree’)	brea caspi	latex: used to seal/caulk canoes, etc.	collected from felled tree	year round
	*Tovomita *sp. 2^c ^[744]	***maja ñi ***(‘tar tree’)	brea caspi	latex: used to seal/caulk canoes, etc.	collected from felled tree	year round
Combretaceae						
	*Buchenavia sericocarpa *Ducke [647]	***nanu ñi***		trunk: construction material	felled	year round
				bark: strips used as a strapping to carry things	stripped from felled tree	year round
Cyclanthaceae						
	*Asplundia *sp. 1^c ^[670]	***noca***		leaves: wrap and cook food in (i.e. fish, fruits, animal intestines, etc.)	cut from plant	year round
Euphorbiaceae						
	*Hevea guianensis *var. *lutea *(Spruce ex Benth.) Ducke & R.E. Schultes [688]	***ejebe ñi***	shiringa	seeds: used to make toy tops for children^d^	collected from ground	unknown
	*Hyeronima alchorneoides *Allemão [727]	***pɨ*** ***pɨdi ñi***	purma caspi	trunk: firewood	felled	year round
				trunk: used to make the hulls, seats, and keels of canoes	felled	year round
	*Nealchornea yapurensis *Huber [711]		fósforo caspi, keresone caspi	trunk: firewood	felled	year round
Fabaceae						
	*Hymenaea palustris* cf. Ducke [701]	***s *** ***o*** ***j *** ***o*** ***ñi***	azúcar huayo	fruits: edible	collected from ground	unknown
				bark: medicinal (rheumatism and paleness)	cut from trunk (not felled)	year round
	*Inga *spp. [660, 661, 699, 708, 761, 764]	***mene ñi***	shimbillo	fruits: edible	picked or collected from cut branches or felled tree	~April-June and October-November
	*Pterocarpus amazonum *(Mart. ex Benth.) Amshoff [739]	***bo come toto ñi ***(‘white paddle buttress tree’)	remo caspi blanco	buttress roots: used to make canoe paddles	cut from buttress root (not felled)	year round
Lauraceae						
	*Aniba *sp. 2^c ^[731]	***bɨ*** ***ya ñi***	muena	trunk: house construction material	felled	year round
				trunk: used to make the hulls, seats, and keels of canoes	felled	year round
	*Endlicheria *sp. 1^c ^[683]	***bɨ*** ***ya ñi***	isma muena	trunk: house construction material	felled	year round
				trunk: used to make the hulls, seats, and keels of canoes	felled	year round
	*Licaria* sp. 2^c^ [740]	***nea bɨ*** ***ya ñi***	cunchi muena	trunk: house construction material	felled	year round
				trunk: used to make the hulls, seats, and keels of canoes	felled	year round
Lecythidaceae						
	*Eschweilera coriacea *(DC.) S.A. Mori [729]	***ɨ*** ***oma ñi, ɨ omɨ ja ñi***	machimango	bark: strips used as a strapping to carry things	stripped from trunk (not felled)	year round
Malvaceae						
	*Theobroma subincanum *Mart. [700]	***ch ocotu ñi*** (‘bald tree’)	cacao amarillo	fruits: edible	collected by climbing or felling tree	~April-June
				bark: processed into a tobacco admixture	cut from trunk (not felled)	year round
	*Theobroma obovatum *Klotzsch ex Bernoulli [723]	***m*** ***e*** ***ch *** ***o*** ***cotu ñi****,* ***m*** ***e*** ***sɨno ñi***	cacaohuillo	fruits: edible	collected from felled tree	~April-June
Marantaceae						
	*Calathea lutea *Schult. [652]	***n *** ***u*** ***ta jao sa***	bijao	leaves: wrap and cook food in (i.e. fish, animal intestines, etc.)	cut from plant	year round
				leaves: wrap and store salt and fariña (a coarse flour or meal made from *Manihot esculenta*) in	same as above	same as above
Meliaceae						
	*Guarea macrophylla *subsp. *pendulistica *(C. DC.) T. D. Penn. [737]	***m *** ***o*** ***j *** ***o*** ***ñi***		trunk: house construction material	felled	year round
Moraceae						
	*Brosimum parinarioides *subsp. *amplicoma *(Ducke) C. C. Berg [663]	***abɨ*** ***yodo ñi***	caucho macho del bajo	latex: used to seal/caulk canoes, etc.	tap trunk	year round
	*Brosimum utile *(Kunth) Oken ex J. Presl [779]	***ayo ñi***	tamamuri	fruits: edible	collected from felled tree	unknown
						
	*Helicostylis scabra *(J.F. Macbr.) C.C. Berg [702]	***yaji ñi***	chimicue	fruits: edible	collected from felled tree	~April-June
	*Naucleopsis glabra *Spruce ex Pittier [717]	***ch *** ***i*** ***cue ñi***		fruits: edible	collected from felled tree	unknown
	*Pseudolmedia laevis *(R. & P.) Macbr. [691]	***naso dei ñi ***(‘*Lagothrix lagothricha*’s *Artocarpus altilis* tree’)	pandisho del mono	fruits: edible	picked	unknown
Myristicaceae						
	*Iryanthera olacoides *(A.C. Sm.) A.C. Sm. [719]	***ɨ mɨ bi t *** ***i*** ***to ñi***	cumala	fruits: edible aril (prepared by wrapping in the leaves of two plant species and heating over fire)	collected from felled tree	~April-June
	*Virola loretensis *A.C. Sm. [732]	***cudu ñi***	cumala	fruits: edible aril (prepared by wrapping in the leaves of two plant species and heating over fire)	collected from felled tree	~April-June
				seeds: used as a fuel for a type of candle	collected from felled tree	~April-June
				trunk: selectively logged and sold^d^	felled	year round
	*Virola pavonis *(A. DC.) A. C. Sm. [775]	***bai cudu ñi*** (‘*Tayassu pecari*’s *Virola *tree’)*, ****miña cudu ñi ***(‘small bird’s *Virola *tree*’*), ***mia cudu ñi ***(‘small bird’s *Virola *tree*’*)	cumala	fruits: edible aril (prepared by wrapping in the leaves of two plant species and heating over fire)	collected from felled tree	~April-June
				seeds: used as a fuel for a type of candle	collected from felled tree	~April-June
				trunk: selectively logged and sold^d^	felled	year round
Ochnaceae						
	*Cespedesia spathulata *(Ruiz & Pav.) Planch. [714]	***ma pede ñi ***(‘red board tree’)		trunk: house construction material	felled	year round
				trunk: firewood	felled	year round
Olacaceae						
	*Minquartia guianensis *Aubl. [758]	***yajisu ñi***	huacapú	fruits: edible	collected from ground	unknown
				trunk: house construction material	felled	year round
Poaceae						
	*Pariana *sp. 1^c ^[644]	***mamecoco***	shacapa, maronilla	leaves: used in shamanic rituals/ceremonies	cut from plant	year round
Rubiaceae						
	*Genipa spruceana *Steyerm. [738]	***be ñi***	huito	fruits: used to dye *Astrocaryum chambira *fibers black	picked	~March-April
Sapotaceae						
	*Chrysophyllum *sp. 1^c ^[741]	***toa ñi ***(‘fire tree’), ***toa acue ñi ***(‘fire fruit tree’)	caimitillo	fruits: edible	collected from felled tree	~January-April
				trunk: firewood	felled	year round
	*Micropholis egensis *(A. DC.) Pierre [721]	***mɨ*** ***catoña ñi***	lagarto caspi	trunk: construction material (used to construct houses, boats, etc.)	felled	year round
				trunk: used to make the hulls, seats, and keels of canoes	felled	year round
				trunk: selectively logged and sold^d^	felled	year round
	*Ecclinusa lanceolata *(M. & E.) Pierre [645]	***toa ñi ***(‘fire tree’), ***toa acue ñi ***(‘fire fruit tree’)	caimitillo	fruits: edible	collected from felled tree	~January-April
				trunk: firewood	felled	year round
Urticaceae						
	*Pourouma cecropiifolia *cf. Mart. [722]	***maca ede ñi***	uvilla del monte	fruits: edible	collected from felled tree	unknown
	*Pourouma cucura *Standl. & Cuatrec.	***maca ede ñi***	uvilla del monte	fruits: edible	collected from felled tree	unknown
	*Pourouma tomentosa *Mart. subsp. *tomentosa *[650]	***maca ede ñi***	uvilla del monte	fruits: edible	collected from felled tree	unknown

See [34] for frequency and density data for useful tree and palm species in sampled plots.

^a ^All specimens, unless otherwise indicated, were collected by M. Gilmore, E. Valderrama, B. Endress, C. Horn, D. Rios Vaca & V. Rios Torres under permit Nº0388-2010-AG-DGFFS-DGEFFS issued by the Ministerio de Agricultura (MINAG), Peru. All voucher specimens are deposited in the Herbarium Amazonense (AMAZ), Universidad Nacional de la Amazonia Peruana, Iquitos, Peru.

^b ^Harvest times are preliminary and approximate, based on consultant testimony and not independently verified by the researchers.

^c ^Numbering of species follows [34].

^d ^Not currently used in this way by the Maijuna of the Yanayacu River basin.

^e ^This specimen was collected by M. Gilmore (with the help of various field assistants) under permit N^o ^71-2003-INRENA-IFFS-DCB issued by the Instituto Nacional de Recursos Naturales (INRENA), Peru. All voucher specimens are deposited in the Herbarium Amazonense (AMAZ), Universidad Nacional de la Amazonia Peruana, Iquitos, Peru and the Willard Sherman Turrell Herbarium (MU), Miami University, Oxford, Ohio.

As previously stated, a wide range of wildlife also eat *M. flexuosa* fruits and, as a result, ***ne cuadu*** become important hunting areas within the Yanayacu River basin from May through August. During these times, the Maijuna hunt in ***ne cuadu*** during both the day and night. To hunt during the night, hunters commonly make hunting platforms close to *M. flexuosa* trees with fruits that show signs of being eaten by animals. They then wait throughout the night with their flashlights and shotguns at the ready. A Maijuna hunter explained how to hunt in ***ne cuadu***:

Yes, you need to make your hunting platform to listen for paca (*A. paca*; ***seme***, ***oje beco***, ***pɨbɨ aco***), armadillos (*Dasypus* sp.; ***toto aquɨ***), and lowland tapirs (*Tapirus terrestris*; ***bequɨ***, ***jaico***) at night. Then they do not smell you fast because you are up above. It also makes it easy to see down below with your flashlight… Black agouti (*D. fuliginosa*; ***maitaco***, ***moñeteaco***, ***codome***), South American coati (*Nasua nasua*; ***chichibɨ***), collared peccary (*Tayassu tajacu*; ***caocoa***, ***yau***), and white-lipped peccary (*Tayassu pecari*; ***sese***, ***bɨdɨ***) come during the day to eat ***ne*** (*M. flexuosa* fruits) and so you can kill them during the day.

****According to consultants, a total of 20 different animal species eat *M. flexuosa* fruit and are hunted in *M. flexuosa* palm swamps. These include, 13 species of mammals, 6 species of birds, and 1 reptile species – all of which are eaten and slightly over half (55%) of which are sold as game meat. These species are listed in Table 2, along with the varied ways that they are used, when they are encountered (day and/or night), and their Maijuna, local and English names. It is also important to note that although Maijuna hunters target *M. flexuosa* palm swamps when *M. flexuosa* is in fruit, they also pass through these areas during other times of the year killing game animals opportunistically.

**Table 2:** **Birds, mammals, and reptiles that, according to Maijuna consultants, eat *M. flexuosa *** **( *ne ñi *) fruit and are hunted in *M. flexuosa *** **palm swamps ( *ne cuadu *) within the Yanayacu River basin**

**Taxon**		**English name**	**Maijuna name**	**Spanish name**	**Time encounter**	**Use**
Birds						
Psittacidae						
	*Ara ararauna*	blue-and-yellow macaw	***bo ma***	*guacamayo amarillo*	day	eat; raise as pets; sell (live birds for pets); used to make fans for fires (feathers); adornment (feathers)^a^
	*Ara chloroptera*	red-and-green macaw	***meme ma***	*guacamayo cabezón*	day	eat; raise as pets; sell (live birds for pets); used to make fans for fires (feathers); adornment (feathers)^a^
	*Ara macao*	scarlet macaw	***ɨ*** ***ma****,* ***gu*** ***ɨ*** ***ma***	*guacamayo rojo*	day	eat; raise as pets; sell (live birds for pets); used to make fans for fires (feathers); adornment (feathers)^a^
	*Ara severa*	chestnut-fronted macaw	***be***		day	eat; raise as pets; sell (live birds for pets); used to make fans for fires (feathers); adornment (feathers)^a^
	*Orthopsittaca manilata*	red-bellied macaw	***ne ɨ*** ***na***	*maracana*	day	eat; adornment (feathers)^a^
Rallidae						
	*Aramides cajanea*	gray-necked wood-rail	***ne t *** ***ɨ*** ***t ɨ***	*unchala*	day	eat
Mammals						
Agoutidae						
	*Agouti paca*	paca	***seme****,* ***oje beco****,* ***pɨ*** ***bɨ aco***	*majaz*	night	eat; sell (meat); used in blowgun construction (teeth used as sightline)^a^
Cebidae						
	*Alouatta seniculus*	red howler monkey	***jaiquɨ****,* ***majei***	*coto mono*	day	eat; sell (meat)
	*Cebus apella*	brown capuchin monkey	***nea t *** ***a*** ***que***	*mono negro*	day	eat
	*Pithecia monachus*	monk saki monkey	***baotutu***	*huapo*	day	eat; used to make a duster (tail)
Cervidae						
	*Mazama americana*	red brocket deer	***bosa****,* ***mɨ *** ***i*** ***bɨ aquɨ***	*venado colorado*	night, day (rarely)	eat; sell (meat); medicinal (antlers); adornment of houses (antlers); used to make drums (hide)
Dasypodidae						
	*Dasypus sp.*	armadillo	***toto aquɨ***	*carachupa*	night	eat; sell (meat); medicinal (tip of tail)
	*Priodontes maximus*	giant armadillo	***jai toto aquɨ***	*carachupa mama*	night	eat; sell (meat); medicinal (claws); adornment of houses (shell of armor plates); used as a container (shell of armor plates)
Dasyproctidae						
	*Dasyprocta fuliginosa*	black agouti	***m *** ***ai*** ***taco****,* ***moñeteaco****,* ***codome***	*añuje*	day	eat; sell (meat); used in blowgun construction (teeth used as sightline)^a^
	*Myoprocta pratti*	green acouchy	***maso***	*punchana*	day	eat; used in blowgun construction (teeth used as sightline)^a^
Procyonidae						
	*Nasua nasua*	South American coati	***chichibɨ***	*achuni*	day	eat; sell (meat)
Tapiridae						
	*Tapirus terrestris*	tapir	***bequɨ****,* ***jaico***	*sacha vaca*	night	eat; sell (meat); medicinal (toenails)
Tayassuidae						
	*Tayassu pecari*	white-lipped peccary	***s *** ***e*** ***s *** ***e****,* ***bɨ*** ***dɨ***	*huangana*	day	eat; sell (meat and hide); used to make drums (hide)
	*Tayassu tajacu*	collared peccary	***caoc*** ***oa****,* ***yau***	*sajino*	day	eat; sell (meat and hide); used to make drums (hide)
Reptiles						
Testudinidae						
	*Chelonoidis denticulata*	yellow-footed tortoise	***meniyo***	*motelo*	day	eat; sell (live tortoises for food); used as a seat (shell); used to make hunting whistles (chest plate from females); medicinal/traditional beliefs (penis)

^a ^Not currently used in this way by the Maijuna of the Yanayacu River basin.

Additionally, like other inhabitants of the Peruvian Amazon, the Maijuna harvest beetle larvae from the trunks of *M. flexuosa* in ***ne cuadu***. However, in addition to harvesting larvae from the beetle *Rhynchophorus palmarum*[3], the Maijuna also harvest the larvae of a second beetle species, *Rhinostomus barbirostris*, as well. These two species of beetle larvae are called ***ne baquɨ*** and ***sañi***, respectively, and are eaten and used as fishing bait year round. Notably, in Maijuna lands, they are subsistence, rather than market, products. ***Ne baquɨ*** (*R. palmarum*), referred to in Spanish by the Maijuna as *surí de aguaje*, requires human intervention to harvest. Maijuna aguaje fruit collectors that have felled *M. flexuosa* trees commonly cut two small holes on either side of the trunk toward the crown of the tree to facilitate cultivation of ***ne baquɨ***. According to Maijuna consultants, this allows the adult of this beetle species, known as ***bɨdico*** in Maijuna, to more easily penetrate the soft parts of the trunk, which it prefers, to lay its eggs. In addition to cutting holes in *M. flexuosa* trees that have been felled for fruit collecting, the Maijuna also cut holes in the trunks of standing juvenile palms. Consultants explained that it is much quicker to do this than to cut down adult palms, and it also helps to prevent giant armadillos (*Priodontes maximus*) from eating the ***ne baquɨ*** as they develop. This ultimately kills the juvenile palm tree. Over all, Maijuna consultants indicated that it takes between 1.5-3 months for ***ne baquɨ*** to reach a size worthy of harvest.

***Sañi*** (*R. barbirostris*), referred to in Spanish by the Maijuna as *surí blanco*, is much different than ***ne baquɨ*** because the Maijuna do not actively cultivate or manage this species. Instead, they opportunistically find these beetle larvae in the trunks of old, naturally fallen *M. flexuosa* trees. According to Maijuna consultants, the adults of this beetle species, known as ***sañi bɨaco*** in Maijuna, prefer to lay their eggs in old, tough tree trunks instead of the younger, softer trunks preferred by ***bɨdico***. Because ***sañi*** are opportunistically harvested, rather than cultivated, they are much less commonly eaten in Maijuna lands than ***ne baquɨ***. It is also important to note that, in addition to eating ***ne baquɨ*** and ***sañi***, the Maijuna also eat the adults (***bɨdico*** and ***sañi bɨaco***) of these two beetle species as well when encountered.

In addition to collecting *M. flexuosa* products in aguajales, the Maijuna also use over 60 different species of plants from these areas. Of the useful plant species found in aguajales, 72% are trees, 22% are palms, and 6% are herbs. They are used in a wide variety of ways, including for construction material, food, and in cultural ceremonies and rituals, among others. Additionally, six species are also used medicinally by the Maijuna to treat a wide variety of conditions, including snakebites, malaria, rheumatism, tuberculosis, paleness, pimples, and abscesses and boils. However, only four species beyond aguaje, all of which are palms (*Astrocaryum chambira* Burret, *Euterpe precatoria* Mart., *Oenocarpus bataua* Mart., and *Socratea exorrhiza* (Mart.) H. Wendl.), are currently market goods with any frequency; the rest (93%) are used for subsistence purposes. Moreover, many of the most abundant species in aguajales are those used by the Maijuna, particularly the palm species [34]. The scientific, Maijuna, and local names of the culturally important plant species found in aguajales, as well as information regarding their use, harvesting method, and time of harvest are detailed in Table 1. This information allows us to not only understand *what* plant species the Maijuna are harvesting from *M. flexuosa* palm swamps but these data also provide us with a much more detailed and integrated knowledge of *how* and *when* they are using these species. This ultimately allows for a much more detailed and nuanced understanding of the use and significance of *M. flexuosa* palm swamps (***ne cuadu***) and their associated resources by the Maijuna.

As detailed, *M. flexuosa* palm swamps (***ne cuadu***) and their associated resources are culturally useful and significant to the Maijuna in a wide variety of ways. In fact, the significance of ***ne cuadu*** to the Maijuna was further highlighted when one of the authors (Gilmore) completed participatory mapping exercises in the different Maijuna communities as part of another project (see [29,30]). During the mapping sessions in each of the Maijuna communities, *M. flexuosa* palm swamps were consistently one of the first things that Maijuna community members chose to map ultimately pointing to the cultural salience and significance of this habitat. Additionally, it was found that 21 different *M. flexuosa* palm swamps within Maijuna ancestral lands have proper Maijuna names, 7 of which are found within the Yanayacu River basin, while there are dozens more that go unnamed. The Maijuna name *M. flexuosa* palm swamps after people, plants, events, and the size or shape of the area, among other things. The extensive naming of *M. flexuosa* palm swamps further highlights their importance to the Maijuna.

In short, *M. flexuosa* palm swamps appear to be perfect examples of what Posey [35] called “resource islands”. Posey ([35]: 117) defines “resource islands” as “…areas in the primary forest where specific concentrations of useful plants or animals are found.” Posey [35] provides several general examples of “resource islands”, including sources of palm hearts, palmito and palm nut sources, areas with cane for arrows, hunting areas, and fish concentrations, among others. According to Posey [35], “resource islands” and their anthropogenic counterparts, “forest-fields”, allowed the Kayapó of the Brazilian Amazon to travel, without relying on domestic agricultural produce, for several months at a time. However, it is unclear whether or not Posey [35] was referring to specific habitats or just areas in general that provided concentrations of resources. Nonetheless, we feel that the concept of “resource islands” can be easily extended to describe *M. flexuosa* palm swamps (***ne cuadu***) and their importance and use by the Maijuna given that ***ne cuadu*** are dominated by the ethnobotanically and economically important palm species *M. flexuosa*. Beyond providing aguaje, we have documented that a wide variety of economically and ethnobotanically important plants are also found in ***ne cuadu***, and that due to the draw of aguaje fruit they are also culturally important hunting areas. Thus, ***ne cuadu*** are islands of multiple resources. Though many of the plants found in ***ne cuadu*** are not restricted to this habitat, the overall frequency and abundance of useful plant species in these areas makes them unique. For example, of the ten most abundant overstory trees in aguajales, five are ethnobotanically or economically important to the Maijuna, further supporting the concept that these ecosystems are “resource islands” [34]. The forests of Amazonia are characterized by very high diversity and generally low frequency of plant species and therefore Maijuna habitats dominated by ethnobotanically and/or economically important plant species, such as ***ne cuadu***, can easily be envisioned as “islands” of resources in a “sea” of otherwise undifferentiated forest (***maca***).

### Traditional beliefs about aguajales

As previously stated there are seven *M. flexuosa* palm swamps within the Yanayacu River basin with proper Maijuna names; one of these is an aguajal that the Maijuna call ***Gogobai ne cuadu***. This *M. flexuosa* palm swamp is named after malevolent female supernatural beings that the Maijuna call ***Gogobai***. According to consultants, these supernatural beings reside in *M. flexuosa* palm swamps, especially large ones. However, it is important to note that although ***Gogobai*** reside in aguajales they also occasionally leave these areas to wander around other parts of the forest to look for prey.

The Maijuna consider ***Gogobai*** to be malevolent for a variety of reasons. For example, according to consultants, ***Gogobai*** sometimes abduct children to bring them to an aguajal so that they can eventually eat them. Although ***Gogobai*** are usually invisible, when presenting themselves to children, they take on the form of a woman that looks like the child’s mother or grandmother in an attempt to lure and deceive the child. The kidnapping of two Maijuna children by a ***Gogobai*** is detailed in the traditional Maijuna story titled “***Gogobaide qu****ɨij****a***” (The story of ***Gogobai***) (Appendix 1). This traditional Maijuna story highlights the dangers that ***Gogobai*** pose to Maijuna children. Ultimately, it reinforces that parents must remain vigilant and alert to protect their children from these malevolent supernatural beings.

In addition to abducting and eating children, ***Gogobai*** also occasionally present themselves to solitary hunters. ***Gogobai*** can cause confusion in these individuals which can ultimately result in them getting lost in the forest. In extreme cases, these individuals never return and are never found. In fact, according to consultants, the *M. flexuosa* palm swamp called ***Gogobai ne cuadu*** in the Yanayacu River basin received its name because three different solitary hunters had run-ins with ***Gogobai*** and each were lost for several days within this particular palm swamp. Interestingly, there are no taboos associated with entering or harvesting resources from this particular, or any, *M. flexuosa* palm swamp and, according to consultants, individuals can hunt and collect in ***Gogobai ne cuadu*** as they see fit which many community members in fact do. It seems that the allure of resources in aguajales overrides any fear that the Maijuna may have of ***Gogobai***.

### Changing times, changing relationships – aguaje and aguajales

The relationship that the Maijuna have with both *M. flexuosa* and *M. flexuosa* palm swamps has changed considerably over the years [2]. For example, aguaje has only been collected for the market economy since the early 1990s. Before this, the Maijuna rarely cut palms for subsistence fruit harvest and/or surí cultivation; instead, most fruit was collected opportunistically from the ground in *M. flexuosa* palm swamps. As a 55-year-old Maijuna individual explained, “In the past, like I said, our ancestors didn’t cut them (*M. flexuosa*) down. They went to aguajales, each one carrying their own basket, and filled them up and collected them (*M. flexuosa* fruits)… No, they didn’t cut them down. Every time they wanted to eat aguaje they went to an aguajal and collected it at the base of trees where it had fallen.” This same individual also stated, “If they did cut [*M. flexuosa*], they would only cut one tree down for personal consumption and to grow and eat surí, nothing more. This is why there was a lot of aguaje [back then]. For example, I only started to cut aguaje [for market] when I was 35 years old (in 1990). This is when I started to cut aguaje.”

During the early 1990s, aguaje changed from a subsistence item to a market good as people from outside communities entered the Yanayacu River basin to both harvest and buy fruit. Outsiders drove the large-scale commercial extraction of aguaje, both by extracting aguaje themselves and by serving as buyers and thus providing market access to Maijuna harvesters, resulting in hundreds and likely thousands of *M. flexuosa* trees being cut down. According to consultants, a high volume of aguaje was collected annually during this time and it was easy to harvest due to its great abundance and ease of access. At times, up to 100 sacks were collected per day. Based on our sampling of 15 sacks of aguaje from Iquitos markets (mean kg of fruit/sack = 33.20, SE = 0.38), this means that approximately 3,320 kg of fruit were, at times, destructively harvested in only one day within the Yanayacu River basin. Not surprisingly, in the 2000s, commercial extraction of aguaje began to decline due to destructive overharvesting of females and the accompanying decline in market access. In fact, all households interviewed (100%) indicated that there has been a decline in *M. flexuosa* abundance and all blame the cutting of females for this trend [2]. As a 57-year-old Maijuna individual explained, “When I was a child there was a lot more aguaje… there weren’t people buying aguaje, there just weren’t any. In approximately the year 1990, buyers from the outside came to look for and buy aguaje. And, people started cutting lots of aguaje and this is how things were destroyed.”

Notably, the Maijuna have not overlooked the impact of destructive harvesting on wildlife. A 58-year-old Maijuna individual stated, “Degraded aguajales do not have strength or power because there is not much food [for animals]… Seeing that there isn’t food, the animals look for other aguajales. Because in degraded aguajales, what can they eat? Nothing, there is nothing for them to eat.” Maijuna concerns about the effects of degraded aguajales on game animals are not surprising when considering that from May 2010 to April 2011, 41% of the communities’ income was generated from the sale of game meat and 93% of households hunted as an income generating activity [2]. Additionally, not only is hunting an important source of income but it is also an important part of Maijuna cultural identity and subsistence. Thus, the Maijuna are not only concerned about the impact that this destructive overharvesting has had on the commercial extraction of aguaje but they are also worried about its effects on game animal populations given the significance of aguajales to Maijuna hunters.

In 2008, the Maijuna restricted access of the Yanayacu River basin to outsiders in an effort to conserve and manage their biocultural resources and to demonstrate to the regional government their desire and interest in establishing a regional conservation area. They identified a desire to develop sustainable economic alternatives for their communities and both Puerto Huamán and Nueva Vida specifically identified aguaje as a resource of interest. A regional consortium (*Proyecto Apoyo al PROCREL*), focused on protected area management and conservation with close ties to the regional government, conducted several aguaje management workshops in Puerto Huamán and Nueva Vida in 2009. A limited number of aguaje harvesters were taught how to climb *M. flexuosa* using harnesses and they also helped the communities to set up aguaje management committees. This resulted in an increased use of non-destructive climbing techniques to harvest aguaje, and in 2010 slightly over half (51%) of all aguaje was harvested via climbing. However, the amount of aguaje harvested in 2010 was just 204 sacks (which is approximately 6,772.8 kg) between the two communities (representing just 5% of their total income from May 2010 to April 2011). This is considerably less than estimates given by community members from when commercial aguaje extraction was at its peak in the 1990s [2]. Ultimately, the current low levels of production as compared to the past are a product of destructive overharvesting, which has resulted in reduced numbers of female palms as well as reduced market access as outside buyers rarely enter the communities to purchase aguaje given that there is no longer a stable resource base [2]. In short, the communities are looking to sustainably increase harvest rates and amounts as well as to enhance and improve their management of both aguaje and aguajales.

It is also important to note that not only has the abundance of *M. flexuosa* declined over the years within the Yanayacu River basin but so has Maijuna traditional knowledge and beliefs [13]. Like many other Amazonian indigenous groups the Maijuna have been culturally influenced and changed over the years by pressure from missionaries, the *patrón* system, regional society, government policies, *mestizos*^c^, and the formal education system, among other things [13,20,21]. For example, although Maijuna schools are bilingual in theory, in practice they emphasize almost exclusively Spanish and teach little about Maijuna history, knowledge, or cultural traditions. Therefore, instead of the curriculum building upon and strengthening Maijuna language and knowledge it instead ignores and marginalizes it. Over the past 50 years, the intensity of converging pressures on Maijuna cultural practices and traditional beliefs has increased in severity and as a result the Maijuna language is endangered, Maijuna cultural practices and traditions (i.e. stories, songs, ceremonies, etc.) are rapidly being lost, and Maijuna traditional biological and ecological knowledge is also rapidly disappearing [13,27]. This has put traditional ecological knowledge and beliefs regarding *M. flexuosa* palm swamps and their associated plant and animal resources at risk. Loss of knowledge and connections to aguajales appears to be changing the relationship between the Maijuna and these areas, and moving to a narrow understanding of these ecosystems as being simply a source of cash income (from *M. flexuosa* fruit) and game meat.

## Conclusions

Through interviews, focus groups, and household surveys we were able to obtain a comprehensive understanding of the long, complex, and detailed relationship that the Maijuna have with aguajales and their associated plant and animal resources. This information is critical to enhance current sustainable harvesting and management efforts targeting aguaje and aguajales in the Yanayacu River basin so that plans can account for the multiple socio-cultural and economic needs of the Maijuna and support the efforts of FECONAMAI in conserving not only ecological systems but also Maijuna cultural traditions. Current sustainable harvesting and management efforts only focus on the commercial harvest of aguaje fruit from aguajales yet, as detailed in this paper, this is only one facet of the relationship that the Maijuna have with this habitat and resource. Moving forward, management plans and any future restoration efforts should take into account other facets of this relationship such as the importance of these areas for game hunting as well as for the extraction of plant resources other than aguaje. Additionally, future efforts should also target the conservation of traditional ecological knowledge and beliefs that the Maijuna have regarding aguajales and their plant and animal resources as this is at great risk of being lost. This would ultimately ensure that both the biological and cultural significance of these areas to the Maijuna is being addressed in a more holistic and comprehensive way.

For example, in conceiving a holistic management plan for the Maijuna, maximizing the economic potential of aguaje fruit harvest would not be the sole objective. Hunting, as both a source of income and as part of Maijuna cultural identity, would necessitate the management of game species in aguajales as another priority objective. Faced with losing traditional ecological knowledge in younger generations and deteriorating ties to the forest, the Maijuna may look on the abundance of cultural resources found in aguajales as an opportune location for rekindling cultural awareness. Thus, while in some communities, prioritizing cultivation and agroforestry systems as a means to sustainably harvest palm resources (e.g. *M. flexuosa*, *A. chambira*, etc.) makes sense to increase economic returns and reduce destructive wild-harvesting, it remains unclear how these types of efforts would affect cultural traditions and relationships with aguajales and other forest systems. While the Maijuna do maintain small agricultural fields, cultivation and agroforestry of *M. flexuosa* have never been a major component of their livelihoods [2,27], thus an increased emphasis on cultivation may have unintended impacts on traditional lifeways and livelihood strategies. Moreover, agroforestry stands may not serve as appropriate hunting grounds, and simply removing aguaje from the aguajal has the potential to weaken connections to nature and cultural traditions by decreasing the use and relevance of aguajal habitats. Thus, the management of wild harvested populations in a manner that promotes ecological, economic, and cultural priorities should be the focus of aguajal management in Maijuna lands.

Given the importance of aguajales in the region surrounding Iquitos, we believe our study provides a much-needed in-depth look at how communities interact with this ecologically, economically, and culturally important habitat. Throughout the region, it is important to more holistically understand the importance of aguajales to local communities to assist in developing multi-use management strategies and support biocultural conservation. Given their complex biology and status as “resource islands”, aguajales are complex biocultural systems and need to be engaged and managed as such. Findings are also relevant to the broader discussion on multi-use tropical forest management, and highlight the need to include more than commercial forest products and ecosystem services (e.g. carbon sequestration) into the research and development of multi-use management plans.

## Endnotes

^a^*Patrones* are colonists and their descendants who exploited indigenous labor to harvest forest resources.

^b^ All Maijuna terms are in bold face italics. Transcription of Maijuna words was accomplished with the help of S. Ríos Ochoa, a literate and bilingual Maijuna individual, using a practical orthography previously established by Velie [36]. The practical orthography developed by Velie consists of twenty-seven letters that are pronounced as if reading Spanish, with the following exceptions: ***ɨ*** is pronounced like the Spanish *u* but without rounding or puckering the lips; ***a***, ***e***, ***i***, ***o***, ***u***, and ***ɨ*** are pronounced like ***a***, ***e***, ***i***, ***o***, ***u***, and ***ɨ*** but nasalized; and in a position between two vowels, ***d*** is pronounced like the Spanish *r*. Also, the presence of an accent indicates an elevated tone of the voice. Accents are only used when the tone is the only difference between two Maijuna words and the word’s meaning is not clarified by its context. The twenty-seven letters that make up the Maijuna alphabet are: ***a***, ***a***, ***b***, ***c***, ***ch***, ***d***, ***e***, ***e***, ***g***, ***h***, ***i***, ***i***, ***j***, ***m***, ***n***, ***ñ***, ***o***, ***o***, ***p***, ***q***, ***s***, ***t***, ***u***, ***u***, ***y***, ***ɨ***, and ***ɨ***.

^c^*Mestizos* are people of mixed Amerindian and Iberian descent found throughout the Peruvian Amazon who practice a mixture of traditional agriculture, hunting, fishing, and forest product extraction for their livelihoods ([37-39] as cited in [40]: 421).

## Appendix 1

English translation of the traditional Maijuna story titled “***Gogobaide quɨija***” (The story of ***Gogobai***). Story told by Samuel Ríos Flores, a master Maijuna storyteller:

“Take care of the children, I am going to the forest to hunt,” [said the father]. “If you go, please return soon. And, why are you talking this way to me about the children?” [said the mother]. “I'm just saying to be very careful with them,” [said the father]. After a while the children wanted to go and swim. “Why are you saying that you want to go and swim? There are demons in those places (in the forest) and you insist that you want to go and swim. Your father was very concerned when he left, he was very concerned when he left and now you want to go and swim,” [said the mother]. The children never got tired of saying, “Mom, we want to go swimming.” “Fine, but first go to fetch water and once collected leave it here, and then you can go to swim,” [said the mother]. And so, the children went to get the water and then brought it to where their mother was. They came to leave [the water] and immediately returned [to the river]…

The children were swimming and laughing. They were laughing while they were swimming. [Then the mother thought,] “What happened to my children, what happened to my children, why aren’t they laughing now,” and she began to call them. She got tired of calling, she got tired of calling. Again…again the children were swimming and jumping into the water. “My grandchildren look over here,” [said ***Gogobai***]. When the children saw her it affected their minds, when they saw her it affected their minds. “Come here, come here,” she called to them. When the children heard this, they went [to ***Gogobai***]. She sat down to allow the children to get into her burden basket. “My grandchildren get in so that I can carry you,” [said ***Gogobai***]. When they heard this, the children climbed in and sat down, and she carried them away. She carried them away and, meanwhile, their mother got tired of calling them because they did not reply.

When the children didn’t respond to her the mother went looking for them [where they were swimming], and she returned, she returned crying. Crying, the mother went running along the same trail that the father took and she started banging on the buttress roots of a tree. When the father heard that she was banging on the buttress roots he started to return [to the house]. “Why is she banging on a buttress root?” he thought. Thinking this he returned. He returned running and got tired. “Why is she banging on a buttress root? Has something happened to my children?” [he asked himself] as he cried while returning. At this moment when he was running a little bird (***chido***) cried out once, yodi yodi yodi yodi. “Why is she banging on a buttress root? Has something happened to my children?” he asked himself again and cried. While he was running he kicked the stump of a tree sapling.

He arrived and found his wife banging on a buttress root. [And, he asked], “Why are you banging on this buttress root?” [She replied,] “What you said would happen to our children before you left, happened. ***Gogobai*** has taken our children.” [And then she asked], “Why do you think so much about [hunting in] the forest?” [He replied], “Didn’t I tell you [that this might happen]? You have to be very careful when they want to go swimming. When I left, I told you that even if you had to scold them you should not let them go swimming.” Upset, he hit her, he hit her until she began to cry.

“I think that I heard them calling before, I heard them,” [said the father]. Upset about the situation he went back [to the forest]. His wife followed him when he was returning [to the forest]. “I heard the screams over there, I heard the screams over there. The children were going down the bank of the creek and they were screaming,” [he said]. He began looking for them… “Where did you hear them when you were returning [home]?” [she asked]. “Let’s go along this side [of the river],” [he said upset with his wife]. They went searching that way and they got tired running. They made a big loop searching the whole riverbank. They looked all over the place but did not find anything.

They then entered another trail and continued [searching]. When they were walking they heard some shouts very far away. “They are yelling from over there. You need to go immediately regardless of how far it is. I'll go by myself and cut a trail so we can find our way back,” [she said]. When he heard this he began to run, and eventually he got tired from running. He climbed up a hill, stopped and did not hear anything… He began to bang on a buttress root very hard, bang on a buttress root, and they (his children) did not answer. After this he went to find his wife… [And, his wife said] “Where did you hear [the screaming] before and why didn’t you listen carefully? I think that I heard them screaming and crying down by the creek.” “How am I supposed to hear well while running?” [he asked]. “Go straight ahead, they were screaming that way. And, don’t think about me, go ahead, I'll cut a trail to meet up with you,” [she said]. He left running for another hill to see if he could hear something, he ran to another hill. He went running up another hill and began to bang hard on a buttress root. He banged hard so that they (his children) could hear him but there was no response. He did everything possible in this area and started to cry.

[He told his wife,] “If you are getting tired then return by yourself and go to sleep. I will not return to meet you until I am able to find them, don’t worry.” [And then she replied,] “I will sleep alone since I do not have my children.” He left his wife and ran to another hill hoping to hear something. He heard the children screaming very far away again. “They are over there, I heard them,” [he thought] and he started to run full speed. “I hope they continue calling,” [thought the father] as he continued running up and over other hills. Then the children became completely silent, they were not screaming any longer. The children were silent, but the father continued his journey. “I heard the screams over there, so I have to continue on in that direction,” [he thought].

The father continued his journey and went down the bank of a creek and suddenly heard the children again. “They are heading in that direction, she (***Gogobai***) is taking them in that direction, she is taking them in that direction,” [he thought]. The father ran up a hill and heard the cries of the children and as he went down the hill he heard their screams even closer. “I hope my children continue screaming,” [he thought] as he was very happy to hear them. The father continued walking very fast…when he was walking he heard his children screaming and he ran after them calling and his children answered him. “Dad, ***Gogobai*** has messed with our heads, Dad, ***Gogobai*** has messed with our heads,” [they said]. “I hope they continue yelling…I hope my children continue yelling,” [the father thought] and every time he heard them he rejoiced. “Dad, ***Gogobai*** wants to eat us, Dad, ***Gogobai*** wants to eat us,” [yelled the children]. They did not stop yelling and as the father was approaching them he continued to hear their screams, when he was approaching them he continued to hear their screams. “Dad, ***Gogobai*** wants to eat us,” [yelled the children].

“Come quickly my grandchildren, let’s go to sleep deeper into the forest, nighttime is quickly approaching,” [said ***Gogobai***]. ***Gogobai*** ran over to the children. “Come over here quickly to my burden basket, come over here quickly to my burden basket,” she said while she sat down for the children to get into her burden basket. When she realized that the children did not want to get into her burden basket she stood in the middle of the creek. “My grandchildren come quickly, it is shallow over here and you can cross so we can go. These *aguaje shambo* (***ma ne***) are for you, come over to the other side of the creek and I will give them to you to eat,” [said ***Gogobai***]. When the youngest child heard this he wanted to cross the creek but his older brother grabbed his hand and prevented him from doing so.

“Dad, ***Gogobai*** wants to eat us,” [yelled the children]. When the father heard this he started to crawl along the ground to remain hidden from ***Gogobai***. “Dad, ***Gogobai*** wants to eat us,” [yelled the children]. “Come quickly my grandchildren, let’s go to sleep deeper in the forest,” [said ***Gogobai***]. Seeing her, the father hid behind a tree and shot her with his blowgun. “Ow, a horsefly is biting me, a horsefly is biting me,” [said ***Gogobai***]. After shooting her the first time with his blowgun, the father did it again. “The horsefly is biting me again,” [said ***Gogobai***]. [Then she said,] “I could have eaten them (the children) a long time ago and now they are the ones that are hurting me. How did they learn to hurt me like this?” And then she fell into the water, she fell into the water and died.

As the father went over to the children they ran towards him. The father began to hug them, he hugged them. The father then picked some leaves of ***mamecoco*** (*Pariana* sp. 1; see Table 1) and used them to cleanse their bodies of any evil (by sweeping or brushing the bundled leaves over their bodies). After the father finished cleansing the children they began to return [home]…and the children told their father, “Dad, she (***Gogobai***) wanted to eat us. She took us by the hands and pulled us towards the creek, we didn’t want to go because we didn’t want her to eat us and that is why we were screaming and crying.” [And then the father said,] “Everything is alright now with her, I know that ***Gogobai*** took you and she wanted to eat you.” While bringing the children [back home], he became upset about what had happened to his children. He brought them home and they arrived very late. “Do you have the children with you?” [the mother asked] and when she saw them she was very happy.

When they arrived the father went to sleep and in his dream he saw ***Gogobai*** and she said to him, “Just as you did me wrong, your children will pay, you are going to lose them. I'm going to take them away from you.” [And, the father said,] “Why are you talking to me like this? If you are saying that you want revenge, take revenge on me and let my children live, take me if you want to but not my children.” …Not far away from the house, there was a shaman. The shaman cured the father when he was on the verge of dying and he got better. If it was not for the shaman, the father would have died. After curing him, the shaman said to him, “You were going to die, you were going to die. You would have been wandering in the forest for eternity. That is what she (***Gogobai***) would have done to you. …Now, you should not think about going to the forest. You have to wait a month before returning to the forest again.” The father did not return to the forest for this time period. The end.

## Competing interests

The authors declare that they have no competing interests.

## Authors’ contributions

MG wrote the manuscript with help from BE and CH. All authors helped to design the study, carry out the field research, and analyze/interpret the data. Data collected during this study was supplemented by data that MG has collected during ethnobiological and ethnoecological field research with the Maijuna since 1999. All authors read and approved the final manuscript.
